# Rhizomes Help the Forage Grass *Leymus chinensis* to Adapt to the Salt and Alkali Stresses

**DOI:** 10.1155/2014/213401

**Published:** 2014-07-13

**Authors:** Xiaoyu Li, Junfeng Wang, Jixiang Lin, Ying Wang, Chunsheng Mu

**Affiliations:** ^1^Key Laboratory of Wetland Ecology and Environment, Northeast Institute of Geography and Agroecology, Chinese Academy of Sciences, Changchun 130102, China; ^2^Key Laboratory of Vegetation Ecology of Ministry of Education, Institute of Grassland Science, Northeast Normal University, Changchun, Jilin Province 130024, China; ^3^Alkali Soil Natural Environmental Science Center, Northeast Forestry University Key Laboratory of Saline-alkali Vegetation Ecology Restoration in Oil Field, Ministry of Education, Harbin 150040, China; ^4^Key Laboratory of Songliao Aquatic Environment, Ministry of Education, Jilin Jianzhu University, Changchun 130118, China

## Abstract

*Leymus chinensis* has extensive ecological adaptability and can grow well in saline-alkaline soils. The knowledge about tolerance mechanisms of *L. chinensis* could be base for utilization of saline-alkaline soils and grassland restoration and rebuilding. Two neutral salts (NaCl : Na_2_SO_4_ = 9 : 1) and two alkaline salts (NaHCO_3_ : Na_2_CO_3_ = 9 : 1) with concentration of 0, 100, and 200 mmol/L were used to treat potted 35-day-old seedlings with rhizome growth, respectively. After 10 days, the biomass and number of daughter shoots all decreased, with more reduction in alkali than in salt stress. The rhizome biomass reduced more than other organs. The number of daughter shoots from rhizome was more than from tillers. Under both stresses, Na^+^ contents increased more in rhizome than in other organs; the reduction of K^+^ content was more in underground than aerial tissue. Anion ions or organic acids were absorbed to neutralize cations. Na^+^ content in stem and leaf increased markedly in high alkalinity (200 mmol/L), with accumulation of soluble sugar and organic acids sharply. Rhizomes help *L. chinensis* to adapt to saline and low alkaline stresses by transferring Na^+^. However, rhizomes lost the ability to prevent Na^+^ transport to aerial organs under high alkalinity, which led to severe growth inhibition of *L. chinensis*.

## 1. Introduction

The salinization-alkalization of soil is an increasing environmental problem and a limiting factor for plant growth and productivity [1]. The problem is still widespread. Of the world's cultivated land, about 0.34 × 10^9^ ha are saline and other 0.56 × 10^9^ ha are sodic; they occupy 23% and 37% of cultivated lands area, respectively [2]. In many inland areas such as northeastern China, alkalinized meadow covers more than 70% of the land area and is expanding. Neutral salts (NaCl and Na_2_SO_4_) and alkaline salts (NaHCO_3_ and Na_2_CO_3_) are the key salt constituents in the soils [3]. Some reports indicated that alkali stress from NaHCO_3_ and Na_2_CO_3_ was more destructive to plants than salt stress from NaCl and Na_2_SO_4_ [1, 4]. For example, the water content of halophyte* Suaeda glauca* shoots was inhibited significantly by 240 mmol/L salt stress or 160 mmol/L alkali stress [5]; the relative growth rate of* Kochia sieversiana* seedlings was inhibited markedly by 100 mmol/L salt stress or 50 mmol/L alkali stress [1]. The effects of salt stress on plants generally involve osmotic stress and ion injury [6]. Alkali stress causes similar effects, adding extra influence of high pH. This stress usually inhibits the absorption of ions such as NO_3_^−^ and SO_4_^2−^, disturbs the ionic balance, and undermines intracellular pH stability [1, 5].

Plants need to maintain osmotic and ion balance by controlling the absorption, transportation, and sequestration of salt ions and synthesizing the organic solutes [7]. Primarily inorganic ions participate in the regulation of ion balance in salt stress, while organic acids are the main components used to balance the excess cations in alkali stress [1, 5, 8]. At the cellular level, plants will lower the uptake and accumulation of Na^+^ in the cytoplasm to avoid the harmful effect of saline ions (especially Na^+^) on cytoplasm. This process is mediated by restriction of Na^+^ influx and/or by active efflux from the cytoplasm to the vacuole and back into the growth medium as well as simultaneous synthesis of micromolecular solutes to balance the osmotic pressure coming from the vacuole [6, 9, 10]. At individual level, plants usually transfer toxic ions (such as Na^+^) to the root or exclude Na^+^ from shoots effectively, to reduce the destructive effects on other organs [11]. Some dicotyledonous halophytes excrete Na^+^ and Cl^−^ using salt glands or bladders [12].

*Leymus chinensis *(Trin.) Tzvel. is a rhizomatous, perennial grass that is widely distributed in the eastern part of the Eurasian steppes including the Russian Baikal, the northern and eastern parts of the People's Republic of Mongolia, the northern and northeast plain of China, and the Loess Plateau of China [13]. It is a dominant species in saline-alkaline soils of the Songnen Plain in northeast of China. It grows rapidly and is highly tolerant to arid and saline-alkaline soils [14].* L. chinensis* is considered one of the most promising grass species for grassland rehabilitation and restoration in northern of China [15, 16]. It has extensive ecological adaptability and plasticity and can grow well in soils with high pH (8.5–11.5) [17]. It also has a high forage value and good palatability, making the grass ideal for rangeland use in arid and semiarid northeastern China [18].

The tolerance mechanisms and adaptive strategies of* L. chinensis* can be the base for utilization of saline-alkaline soils and grassland restoration and rebuilding. Rhizomes of* L. chinensis* are special underground organs which function in vegetative reproduction, nutrient storage [19]. and growth adjustment. Genetics and organogenesis of* L. chinensis* were studied [20, 21]. However, little information is available about physiological changes and saline-alkaline tolerance of* L. chinensis*. We propose that the existence of rhizome helps* L. chinensis *to adapt to salt and alkali stresses. To test this hypothesis, the effects of salt and alkali stress on the growth, physiological responses characteristics, and adaptive strategies of* L. chinensis* seedlings were compared and determined the contribution of rhizome to the saline- or alkaline-tolerance. Based on the previous research of salt stress and alkali stress effects on halophytes, 0–200 mmol/L levels were chosen to treat* L. chinensis* seedlings in the experiment. Natural saline-alkaline soils are very complex systems; therefore, the physiological responses and adaptive strategies were studied in sandy culture experiments, simulating salt or alkali stresses with adding neutral or alkaline salts.

## 2. Materials and Methods

### 2.1. Plant Material

The experiment was performed at Northeast Normal University (Jilin Province, China). Mature* L. chinensis *seeds (with a thousand-seed weight of 2.35 g) were collected from the Grassland Ecosystem Field Station at the Institute of Grassland Science, Songnen Grassland, Jilin Province, China (123°44′E, 44°44′N, 167 ma.s.l.) in July 2008. The field station is a semiarid, continental monsoonal climate with a frost-free period of about 140 days; annual average temperature is 4.9°C and annual precipitation is 360 mm (1999–2008) [22]. The soil pH was 8.4 [23]. The harvested seeds were then stored in paper bags at 4°C until the experiments were carried out in May 2009.

Seeds with a germination rate of over 80% were sown in 21 cm diameter plastic pots containing washed sand. Seven holes were drilled in each pot and 4–6 seeds were sown in each hole. All pots were watered with half-strength Hoagland's nutrient solution daily after the emergence of seedlings. The Hoagland solution consisted of 5.0 mmol/L Ca^2+^, 2.0 mmol/L Mg^2+^, 6.0 mmol/L K^+^, 22.2 *μ*mol/L (EDTA)-Fe^2+^, 6.7 *μ*mol/L Mn^2+^, 3.16 *μ*mol/L Cu^2+^, 0.77 *μ*mol/L Zn^2+^, 2.1 mmol/L SO_4_^2−^, 1.0 mmol/L H_2_PO_4_^−^, 46.3 *μ*mol/L H_3_BO_3_, 0.56 *μ*mol/L H_2_MoO_4_, and 15.0 mmol/L NO_3_^−^ [5].

### 2.2. Stress Conditions and Treatments

A 9 : 1 molar mixture of two neutral salts (NaCl : Na_2_SO_4_) or two alkaline salts (NaHCO_3_ : Na_2_CO_3_) was added to half-strength Hoagland's nutrient solution and used for salt and alkali stress treatment groups, respectively. Two concentrations (100 and 200 mmol/L) were applied to each stress group, that is, S100, S200, A100, and A200, and the control (C) received half-strength Hoagland's nutrient solution without salts. The pH ranges in the control, salt, and alkali stress groups were 6.27, 6.43–6.48, and 9.15–9.17, respectively.

When the seedlings were 20 days old, the seedlings were thinned to one plant per hole and each pot contained a total of seven similar sized seedlings. Fifteen pots (5 groups, 3 replications) were placed outdoors and protected from rain. All plants per pot would be divided into leaves, stems, rhizomes, and roots, for determining inorganic ions and organic solutes after grounding. Stress treatments were applied daily (17.00-18.00 h) to 35-day-old seedlings using a total of 500 mL of nutrient solution containing the appropriate stress salts per pot. Controls were treated with the same volume of nutrient solution. The duration of treatment was 10 days, which was when plant leaves in the highest concentration of alkali treatments began to yellow and wilt.

### 2.3. Growth and Vegetative Reproduction

**Figure 1:**
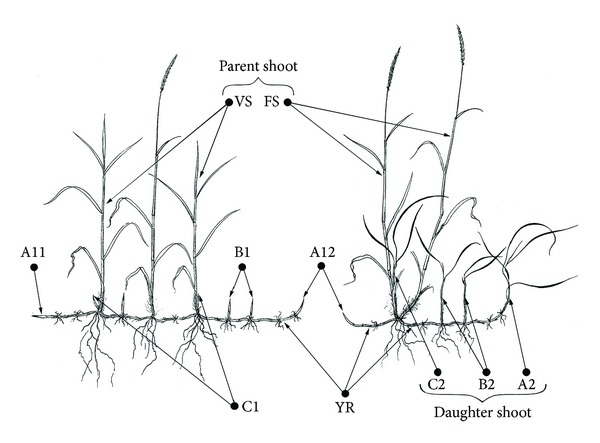
General morphology of Leymus chinensis. Abbreviations: A11, horizontal apical rhizome bud; A12, vertical apical rhizome bud; A2, daughter apical rhizome shoot; B1, axillary rhizome bud; B2, daughter axillary rhizome shoot; C1, shoot bud; C2, daughter axillary shoot; FS, parent flowering shoot; VS, parent vegetative shoot; YR, young rhizome [25]. Here we considered A11, A12, A2, B1, and B2 as the daughter shoots from rhizomes (DSR), C1 and C2 as the daughter shoots from tillers (DST); all these young shoots are included in the total number of daughter shoots (TDN), and YR as rhizome number (RN).

All plants were harvested in the morning after final treatment and washed with tap water followed by three washes with distilled water. We recorded the number of daughter shoots from tiller (DST), the number of daughter shoots from rhizome (DSR), the rhizome number (RN), and total number of daughter shoots (TDN) in each pot (Figure 1). The rhizome, root, stem, and leaf tissue were separated and oven-dried at 105°C for 15 min. Samples were then dried at 70°C to reach a constant weight, and the weights were recorded (DW).

### 2.4. Inorganic Ions

Dry samples (50 mg of each tissue type) were treated with 10 mL deionized water at 100°C for 1 h, and the extracts were used to determine the inorganic ions and organic solutes.

An atomic absorption spectrophotometer (TAS-990, Purkinje General, Beijing, China) was used to determine the Na^+^, K^+^, free Ca^2+^, and free Mg^2+^ content. NO_3_^−^, Cl^−^, SO_4_^2−^, and H_2_PO_4_^−^ were measured by ion chromatography (DX-300 ion chromatographic system, DIONEX, Sunnyvale, USA) using a AS4A-SC ion-exchange column, a CDM-II electrical conductivity detector, and a mobile phase of Na_2_CO_3_ : NaHCO_3_ = 1.7 : 1.8 mM.

### 2.5. Organic Solutes

Proline and soluble sugar (SS) content were estimated spectrophotometrically using ninhydrin and anthrone, respectively [24]. The organic acid (OA) components (citrate, malate, acetate, succinate, and oxalate) were determined by ion chromatography (DX-300 ion chromatographic system, DIONEX, Sunnyvale, USA) using an ICE-AS6 ion-exclusion column (20°C), a CDM-II electrical conductivity detector, a AMMS-ICE II suppressor, a mobile phase with 0.4 mM heptafluorobutyric acid, a flow rate of 1.0 mL/min, and an injection volume of 50 *μ*L. All sample solutions were filtered through a 0.22 *μ*m filter before analysis. Ultrapure water was used throughout the study. The total OA content was calculated based on cumulative values for the five OA components.

### 2.6. Statistical Data Analysis

The data were analyzed by one-way factorial ANOVA using the statistical program SPSS 13.0 (SPSS Inc., Chicago, IL, USA). The means and standard errors are reported. Different salt levels were compared using the Least Significant Difference multiple comparison test and different stress treatments were compared by *t*-tests. Significance level was set at *P* < 0.05.

## 3. Results

### 3.1. Organ Biomass

With increasing solute concentration, the dry weight of all organs decreased significantly (*P* < 0.05; Table 1) under both stresses. Dry weight reduction was much higher under alkali treatment than under salt stress. Compared to the controls (0.24, 0.16, 0.05, and 0.05 mg/plant), A200 reduced the dry weight of leaves (66.7%), stems (62.5%), rhizomes (60.0%), and roots (60.0%). S200 decreased the dry weight of leaves (33.3%), stems (37.5%), rhizomes (40.0%), and roots (20.0%).

### 3.2. Vegetative Reproduction

Under both stresses, vegetative reproductive indices were all markedly lower as the concentration increased (*P* < 0.05; Figure 1 and Table 1). Compared to the controls (10.43, 7.57, 2.86, and 3.10/plant), A200 treatment decreased the TDN (68.0%), DSR (74.8%), DST (50.0%), and RN (44.6%), while S200 decreased the TDN (39.7%), DSR (42.1%), DST (33.3%), and RN (26.2%).

### 3.3. Inorganic Ions

**Figure 2:**
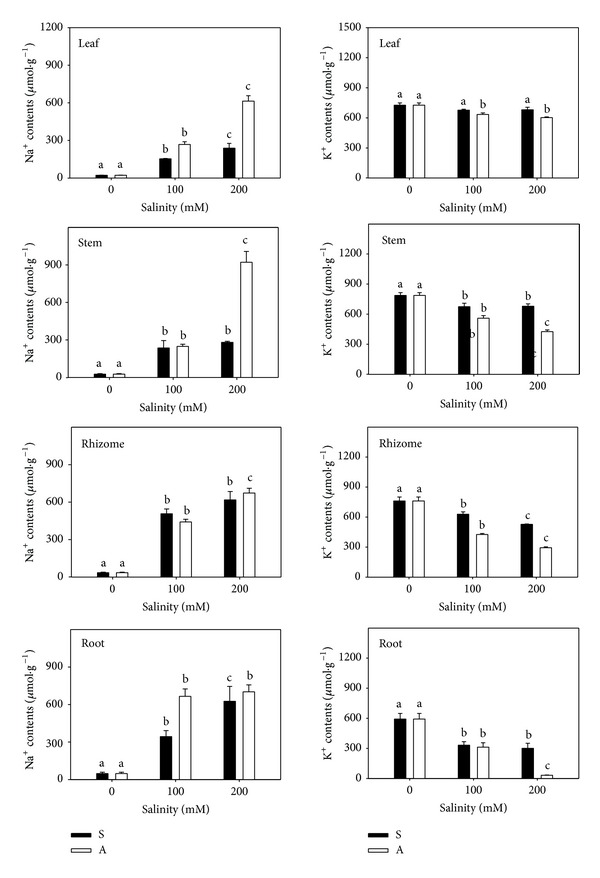
The Na^+^ and K^+^ contents. The effects of salt stress (S) and alkali stress (A) on Na^+^ and K^+^ contents in rhizomes, stems, roots, and leaves of* L. chinensis*. There are three levels under both stresses: 0, 100, and 200 mmol/L. The means and standard errors are reported. Within each column, the different letters indicate significant difference among treatments (*P* < 0.05).

The Na^+^ content in all organs increased sharply with the increasing concentration under both stresses (Figure 2). Under salt stress, the Na^+^ content in leaf, stem, rhizome, and root increased 9.8-, 9.3-, 16.6-, and 12.2-fold compared to the controls (22.03, 27.39, 35.06, and 47.54 *μ*mol/g); Na^+^ increased more in rhizome and root than in stem and leaf. The concentration of Na^+^ was higher in rhizome and root than in stem and leaf at A100, while Na^+^ level was higher in leaf (26.8-fold) and stem (32.6-fold) than in rhizome (18.2-fold) and root (13.8-fold) at A200 (Figure 2). The Na^+^ content was not significantly different between salt and alkali stresses in any of the organs except the root at 100 mmol/L. But the Na^+^ content in stem and leaf was much higher at A200 than at S200 (*P* < 0.05; Figure 2).

The level of K^+^ in all organs decreased significantly. Under salt stress, the K^+^ decrease in root (30.8%) and rhizome (49.2%) was much higher than in stem (13.7%) and leaf (6.3%) compared with the control (726.51, 786.59, 761.56, and 593.40 *μ*mol/g). Under alkali stress, the K^+^ decrease was notably higher for root (94.6%) and rhizome (61.5%) than stem (45.9%) and leaf (17.1%). There was no significant difference in the K^+^ level of all organs between S100 and A100, but the decrease of K^+^ was higher at A200 than at S100 (*P* < 0.05; Figure 2).

**Figure 3:**
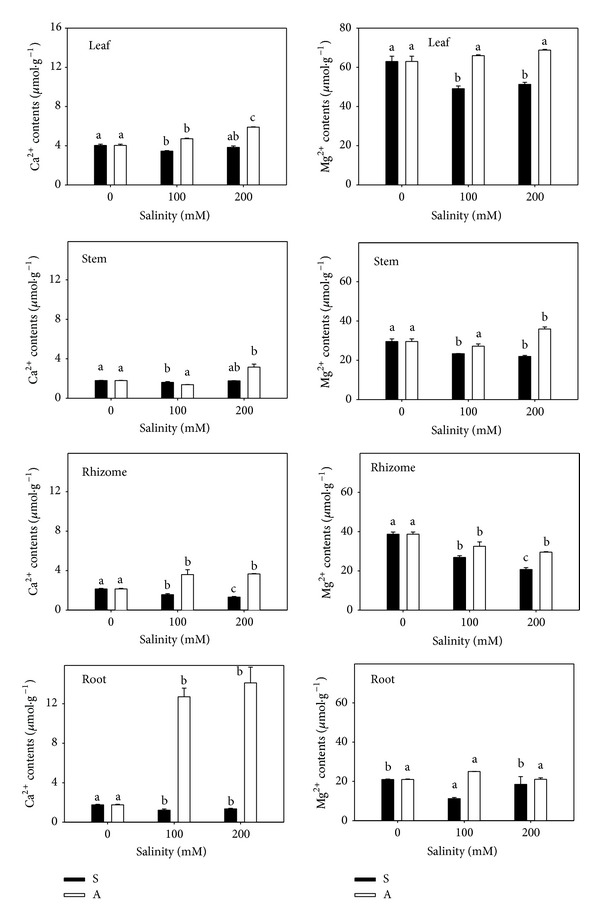
The Ca^2+^ and Mg^2+^ contents. The effects of salt stress (S) and alkali stress (A) on the Ca^2+^ and Mg^2+^ contents in rhizomes, stems, roots, and leaves of* L. chinensis*. There are three levels under both stresses: 0, 100, and 200 mmol/L. The means and standard errors are reported. Within each column, the different letters indicate significant difference among treatments (*P* < 0.05).

Ca^2+^ content decreased under salt stress but increased significantly under alkali stress. Compared to the controls (4.04, 1.79, 2.15, and 1.76 *μ*mol/g), the Ca^2+^ content of leaf, stem, rhizome, and root increased 46.0%, 75.8%, 71.2%, and 700%, respectively. Roots had the greatest accumulation of Ca^2+^ under alkali stress. The Mg^2+^ content decreased significantly (Figure 3) in all organs under salt stress, but it only decreased in rhizomes under alkali stress, with no change in leaf and root and a small increase in stems.

**Figure 4:**
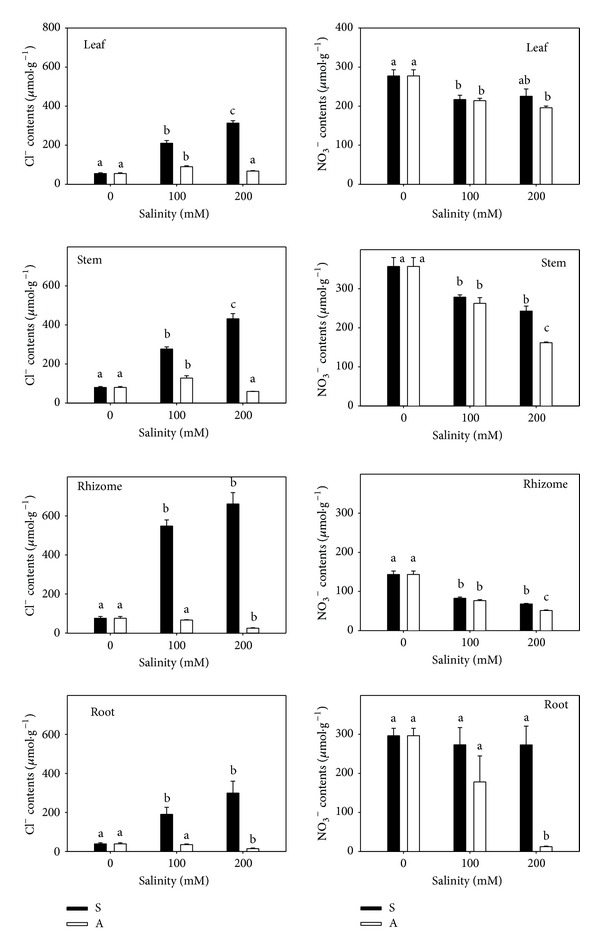
The Cl^−^ and NO_3_^−^ contents. The effects of salt stress (S) and alkali stress (A) on the Cl^−^ and NO_3_^−^ content in rhizomes, stems, roots, and leaves of* L. chinensis*. There are three levels under both stresses: 0, 100, and 200 mmol/L. The means and standard errors are reported. Within each column, the different letters indicate significant difference among treatments (*P* < 0.05).

With increasing concentration, the Cl^−^ content increased sharply in all organs under salt stress (Figure 4). The increase was higher in root (6.6-fold) and rhizome (7.8-fold) than in leaf (4.7-fold) and stem (4.4-fold). Under alkali stress, the Cl^−^ content changed little compared to salt stress (Figure 4).

The NO_3_^−^ content decreased significantly under both stresses. Under salt stress, the NO_3_^−^ content in rhizome (52.4%) decreased more than in root (7.9%), stem (32.0%), and leaf (18.8%) compared with the control (277.44, 356.84, 143. 43, and 296.47 *μ*mol/g). Under alkali stress, the underground organs root (95.8%) and rhizome (64.2%) had greater reductions in NO_3_^−^ than did stem (54.7%) and leaf (29.4%).

**Figure 5:**
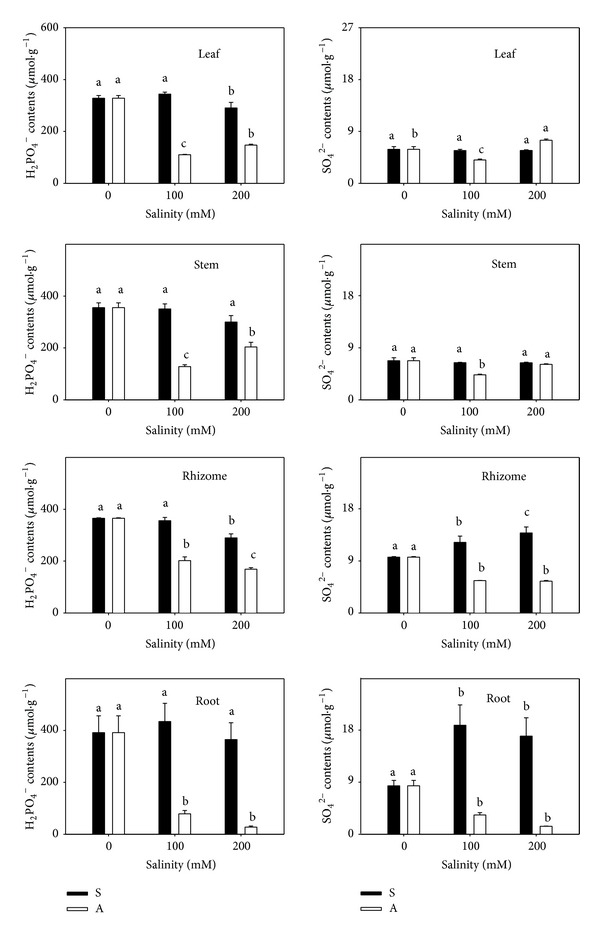
The H_2_PO_4_^−^ and SO_4_^2−^ contents. The effects of salt stress (S) and alkali stress (A) on H_2_PO_4_^−^ and SO_4_^2−^ content in rhizomes, stems, roots, and leaves of* L. chinensis*. There are three levels under both stresses: 0, 100, and 200 mmol/L. The means and standard errors are reported. Within each column, the different letters indicate significant difference among treatments (*P* < 0.05).

The effect on H_2_PO_4_^−^ and SO_4_^2−^ differed between the two stresses. H_2_PO_4_^−^ content changed little under salt stress, but it decreased significantly in all organs under alkali stress. The H_2_PO_4_^−^ decrease in root (93.0%) was greater than in rhizome (53.8%), stem (42.6%), and leaf (55.2%) compared with the control (328.04, 355.32, 365.22, and 391.56 *μ*mol/g). The SO_4_^2−^ content increased markedly under salt stress (Figure 5), but it was reduced by alkali stress (*P* < 0.05).

### 3.4. Organic Solutes

**Figure 6:**
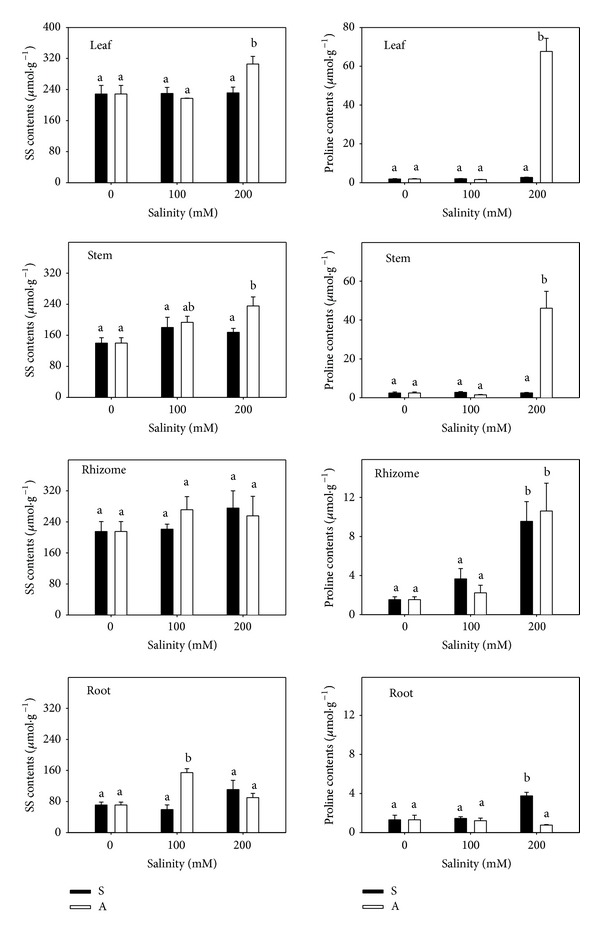
The SS and proline contents. The effects of salt stress (S) and alkali stress (A) on the SS and proline content in rhizomes, stems, roots, and leaves of* L. chinensis*. There are three levels under both stresses: 0, 100, and 200 mmol/L. The means and standard errors are reported. Within each column, the different letters indicate significant difference among treatments (*P* < 0.05).

The SS contents did not change significantly under salt stress, but they increased slightly under alkali stress (Figure 6). The proline content changed with the stress type, concentration, and plant organ. Neither S100 nor A100 induced the accumulation of proline. However, proline was accumulated in some organs under S200 and A200. Under salt stress, only rhizome and root significantly accumulated proline (*P* < 0.05; Figure 6), and the increase was 5.2- and 1.9-fold greater than the controls (1.54 and 1.32 *μ*mol/g). Under alkali stress, proline accumulated sharply in leaf, stem, and rhizome, and the increase was 35.2-, 18.0-, and 5.9-fold compared to controls (1.87, 2.42, and 1.54 *μ*mol/g). The proline content under alkali stress was far higher than under salt stress.

**Figure 7:**
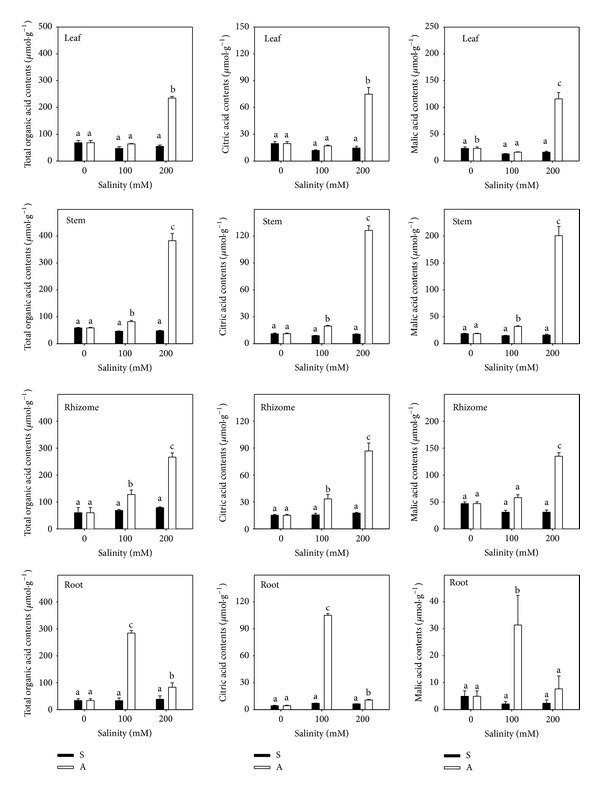
The generous organic acids content. The effect of salt stress (S) and alkali stress (A) on total organic acids, citric acid, and malic acid contents in rhizomes, stems, roots, and leaves of* L. chinensis*. There are three levels under both stresses: 0, 100, and 200 mmol/L. The means and standard errors are reported. Within each column, the different letters indicate significant difference among treatments (*P* < 0.05).

**Figure 8:**
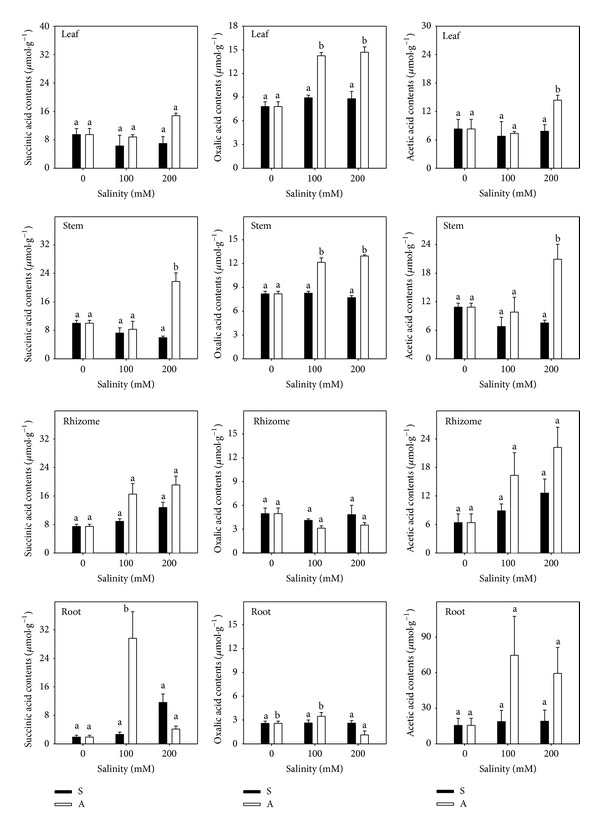
The micro organic acids components contents. The effect of salt stress (S) and alkali stress (A) on the succinic acid, acetic acid, and oxalic acid contents in rhizomes, stems, roots, and leaves of* L. chinensis*. There are three levels under both stresses: 0, 100, and 200 mmol/L. The means and standard errors are reported. Within each column, the different letters indicate significant difference among treatments (*P* < 0.05).

Total OA did not change significantly under salt stress (*P* > 0.05; Figure 7) but accumulated markedly under alkali stress (*P* < 0.05; Figure 7). The increase in OAs in leaf, stem, rhizome, and root was 0-, 0.4-, 1.1-, and 7.4-fold at A100 and 2.4-, 5.5-, 3.4-, and 1.5-fold at A200 in comparison to the controls (68.54, 58.74, 60.17, and 33.70 *μ*mol/g). With increasing alkali treatment concentration, the site of OA accumulation changed from root to leaf. In roots, OA accumulation peaked at A100 and decreased at A200. This pattern differed from rhizome, stem, and leaf, which had increased OA accumulation with increased alkalinity. Like total OA, OA components were unchanged under salt stress, but they accumulated significantly under alkali stress (Figures 7 and 8). Malate and citrate were the key OA components in all organs, and their concentration was highest in the A100 treatment in roots.

## 4. Discussion

The effects of salt and alkali stresses on organs biomass and vegetative reproduction were different. No significant difference was found between S100 and S200, but the biomass and vegetative reproduction reduced higher at A200 than at A100. The results indicated that the growth tolerances of* L. chinensis *to salt stress and alkali stresswere similar at 100 mmol/L and separated at 200 mmol/L. The phenomenon was in accordance with the photosynthesis activity in our previous study [26], which proved that there existed more destructive effects of alkali stress than salt stress.

The salt-tolerance of the aerial and underground organs differed. Some studies indicated that the shoot was more sensitive to salt than the root, as was also seen in young umbu plants [27], but in maize [28] and wheat [29], the root was more sensitive than shoot. In our study, both salt stress and alkali stress reduced the organ biomass of* L. chinensis* significantly. Decrement in rhizome weight was the largest of all organs surveyed, indicating that the rhizome was more sensitive than the other organs. Rhizome in* L. chinensis *bore the brunt of damage from the alkali or salt, sharing the negative effects of salt and alkali stress on other organs for* L. chinensis* growing.

*L. chinensis* was a rhizomatous and perennial forage grass, which formed populations by clonal reproduction of tiller nodes and rhizomes. The daughter plants formed from the tiller nodes and rhizomes were important components of* L. chinensis* populations. During the growing season, the tiller nodes and rhizomes had buds that germinated, which we designated daughters shoots from tillers (DST) and daughters shoots from rhizomes (DSR) (Figure 1). Salt stress usually caused a considerable reduction in tiller and rhizome number in rhizomatous forage grasses, such as* Spartina alterniflora *Loisel [30]. A similar result was found in our experiments. DSR, DST, and RN decreased in salt and alkali stress significantly. These results indicated that the clonal organs of* L. chinensis* were affected by both types of stress, leading to a decrease in vegetative reproduction (Table 1). The reduction of clonal reproduction in* L. chinensis* might allow an individual to be able to alleviate and resist the harmful effects of salt and alkali stress. The more decrement of DSR than DST indicated that the capacity for* L. chinensis* population expansion by rhizome was more affected than population expansion by tiller under salt stress.

Na^+^ was a key toxic cation of salt stress, and neither halophytes nor glycophytes could tolerate large amounts of Na^+^ in the cytoplasm. Under saline conditions, they either restricted the excess Na^+^ in the vacuole or compartmentalized the ions into different tissues to facilitate their metabolic functions [31, 32]. The compartmentalization of Na^+^ was an essential mechanism used by all plants rather than the evolution of tolerance of enzymatic functions in plants from saline environments [11]. Parida and Das (2005) [31] indicated that one of the mechanisms of salt tolerance was the control of ion uptake by roots and transport into leaves. Such as, wheat accumulated Na^+^ mainly in roots, which reduced the harmful effect of Na^+^ on shoots [33]. When* L. chinensis* seedlings grew under salt or low alkali stresses, the Na^+^ content was higher in root and rhizome than that in stem and leaf. This phenomenon indicated that* L. chinensis* might retain excess Na^+^ in the rhizome and root and control Na^+^ transport to stem and leaf. In saline soils, Na^+^ that entered root cells in the outer part of root was likely pumped back out again via plasma membrane Na^+^/H^+^ antiporters encoded by the gene SOS1 [34]. High sodic salt levels induced the expression of amiloride-resistant Na^+^/H^+^ antiporter that could account for the remarkable tolerance to NaCl [35]. High pH condition of alkali stress led to the lacking of H^+^ in the outer part of root in* L. chinensis* seedlings. Therefore, the high levels of Na^+^ and lacking of H^+^ around* L. chinensis* root resulted in a high Na^+^ and H^+^ concentration gradients existing between intracellular and extracellular parts of root, which made it easier for Na^+^/H^+^ antiporters to export H^+^ and import excess Na^+^. With increasing alkalinity, the Na^+^ and H^+^ concentration gradients increased. Thus, sharp increase of Na^+^ in* L. chinensis* root happened (Figure 2). Excess Na^+^ absorption in root exceeded the carrying capacity of root and rhizome and led to the sharp increases of Na^+^ in stem and leaf.

Plant accumulation of Na^+^ can reduce the absorption of other cations. Dudeck and Peacock (1993) reported that Na^+^, Mg^2+^, K^+^, and Ca^2+^ competed for absorption in several paspalum plants they studied [36]. Nutrient deficiency can be caused by salt stress due to competition between Na^+^ and other nutrients. In our study, the absorption of Na^+^ only competed with K^+^ under both stresses. Ca^2+^ kept being unchanged under salt stress, even increased under alkali stress. The transient Ca^2+^ increase potentiated stress signal transduction and led to salt adaptation [37].* L. chinensis* accumulated Ca^2+^ significantly under alkali stress (Figure 3), indicating that Ca^2+^ might be a unique signal response of* L. chinensis* to high pH.

Plants usually accumulated inorganic anions (e.g., Cl^−^, NO_3_^−^, SO_4_^2−^, or H_2_PO_4_^−^) to balance excess Na^+^ [38]. Na^+^ and Cl^−^ were much cheaper source of osmotic solute because ion uptake and accumulation require less energy compared to synthesis of organic solutes [39]. The content and increment of Cl^−^ in rhizomes were far higher than in other organs, indicating that Cl^−^ was one of main physiological response solutes. As the key nutritional sources for plants, NO_3_^−^ and H_2_PO_4_^−^ played important roles in plant growth. However, both stresses inhibited their absorption, especially alkali stress. Alkali stress severely restricted the absorption of nutrient elements such as N and P (Figures 4 and 5). Among the vegetative organs, rhizome had the highest accumulation of Na^+^ and Cl^−^ and the lowest levels of NO_3_^−^ and K^+^, followed by roots. The presence of the rhizome reduced the injury on root, stem, and leaf caused by salt and alkali stress.

A component of the cellular compartmentation model of salt tolerance at the cellular level was the need for the accumulation of metabolically “compatible” (organic) solutes in the cytoplasm to balance the osmotic potential of the Na^+^ and Cl^−^ accumulated in the vacuole [40]. These compatible solutes included mainly proline, glycine betaine, sugars, and polyols [31]. Under salt and alkali stresses, the SS content did not change significantly, and we therefore concluded that SS were not the most important response solutes for osmotic adjustment in* L. chinensis*. Proline was one of amino acids that distributed in plants widely and it accumulated in higher amounts than other amino acids in salt-stressed plants [41, 42]. Proline acted as a signalling/regulatory molecule able to activate multiple responses that were components of adaptation to abiotic stress including salt stress [43]. Some reports argued that increased proline under stress is a product of but not an adaptive response to stress [44, 45]. The proline content did not increase at low concentrations, but it increased at high concentrations under salt and alkali stress, indicating that the accumulation of proline did not occur in response to salt stress but was a product of damage from salt stress (Figure 6).

The deficit of negative charge and ion imbalance under alkali stress caused plants to accumulate organic acids to balance the excess cations and supply the negative charge; thus an increase in OA was a physiological response of alkaline-tolerant plants to alkali stress [1, 25, 29]. OA metabolism played a key role in adjusting stable pH, which could decrease the cellular water potential and increased the activity of mechanisms to avoid cell physiological drought [46]. The OA of* L. chinensis* only increased significantly under alkali stress, especially in stem and leaf at 200 mmol/L. The phenomenon corresponded to excess positive charge caused by sharp increase in Na^+^ (Figure 7).

Under alkali stress, Chenopodiaceae halophytes such as* Suaeda glauca* accumulated oxalate, which comprised more than 90% of total OA [5]. Graminaceous halophytes such as* Puccinellia tenuiflora* accumulated citrate, which comprised more than 90% of total OA [47]. In* L. chinensis*, malate and citrate were the most abundant OA components in all organs, indicating that plants used different mechanisms of OA metabolism under alkali stress. The proportion of malate and citrate to total OA in leaf, stem, rhizome, and root was 81.3%, 85.5%, 83.2%, and 55.9%, respectively (Figure 8). Similar to total OA, malate and citrate increased then decreased in root but increased in other organs with increasing alkalinity. This change of OA contents in root might be related to the root injury. The OA metabolism of different organs* L. chinensis* under alkali stress was still not sure and needed further study.

Our research discussed the growth and physiological responses of vegetative organs in* L. chinensis* seedlings to short time stresses (10 days) caused from salt or alkali conditions. Inorganic ions distribution and organic solutes accumulation in different organs of* L. chinensis* seedlings showed the tolerance and adaptive strategies, especially in rhizomes. The effect of long exposure to saline or alkaline conditions on productivity and forage quality of* L. chinensis* should be researched in the future.

## 5. Conclusion

*L. chinensis* was considered one of the most promising grass species for grassland rehabilitation and restoration in northern of China. That is because it was a rhizomatous, perennial grass. From the results of our study, the salt tolerance of the aerial and underground organs differed in* L. chinensis *seedlings. Rhizome was more sensitive than the other organs, which had the highest accumulation of Na^+^ and Cl^−^ and the lowest levels of NO_3_^−^ and K^+^, followed by roots. At the same time, the more decrement of DSR than of DST indicated that the capacity for* L. chinensis* population expansion by rhizome was more affected than population expansion by tiller under salt stress. The presence of the rhizome reduced the injury on root, stem, and leaf caused by salt and alkali stress.

SS were not the most important response solutes for osmotic adjustment in* L. chinensis*. Proline was a product of damage from salt stress. The significant accumulation of OA in stem and leaf at 200 mmol*·*g^−1^ corresponded to excess positive charge caused by sharp increase in Na^+^. Malate and citrate were the most abundant OA components in all organs, indicating that plants used different mechanisms of OA metabolism under alkali stress.

## Figures and Tables

**Table 1:** The effects of salt stress (S) and alkali stress (A) on biomass and vegetative reproduction of* L. chinensis*.

Stress category	Concentration	Biomass (means ± SE, mg)				Vegetative reproduction (means ± SE,/plant)			
		Leaf	Stem	Rhizome	Root	DSR	DST	RN	TDN
Salt stress	C	0.24 ± 0.003^a^	0.16 ± 0.004^a^	0.05 ± 0.004^a^	0.05 ± 0.007^a^	7.57 ± 0.541^a^	2.86 ± 0.218^a^	3.10 ± 0.048^a^	10.43 ± 0.378^a^
	S100	0.17 ± 0.007^b^	0.11 ± 0.005^b^	0.04 ± 0.005^b^	0.05 ± 0.006^a^	5.38 ± 0.497^b^	1.90 ± 0.265^b^	2.81 ± 0.454^a^	7.29 ± 0.756^b^
	S200	0.16 ± 0.018^b^	0.10 ± 0.012^b^	0.03 ± 0.003^c^	0.04 ± 0.002^b^	4.38 ± 0.172^b^	1.90 ± 0.126^b^	2.29 ± 0.000^b^	6.29 ± 0.286^b^

Alkali stress	C	0.24 ± 0.003^a^	0.16 ± 0.004^a^	0.05 ± 0.004^a^	0.05 ± 0.007^a^	7.57 ± 0.541^a^	2.86 ± 0.218^a^	3.10 ± 0.048^a^	10.43 ± 0.378^a^
	A100	0.19 ± 0.012^b^	0.12 ± 0.008^b^	0.04 ± 0.007^b^	0.04 ± 0.004^b^	5.86 ± 1.072^a^	1.53 ± 0.126^b^	2.38 ± 0.290^b^	7.38 ± 1.061^b^
	A200	0.08 ± 0.008^c^	0.06 ± 0.005^c^	0.02 ± 0.002^c^	0.02 ± 0.003^c^	1.90 ± 0.265^c^	1.43 ± 0.082^b^	1.71 ± 0.143^c^	3.33 ± 0.208^c^

The means and standard errors are reported. C: without salt or alkali stresses, S100: salt stress with 100 mmol/L concentration, S200: salt stress with 200 mmol/L concentration, A100: alkali stress with 100 mmol/L concentration, and A200: alkali stress with 200 mmol/L concentration. In the same stress category, within each column, the different letters indicate significant difference among treatments (*n* = 3, *P* < 0.05). DSR: daughter shoots from rhizome; DST: daughter shoots from tillers; RN: rhizome number; TDN: total daughter number.

## Abbreviations

DST: Daughter shoot from tiller
DSR: Daughter shoot from rhizome
RN: Rhizome number
TDN: Total daughter shoot number
SS: Soluble sugar
OA: Organic acid
C: Control.
